# Expanding our understanding of the trade in marine aquarium animals

**DOI:** 10.7717/peerj.2949

**Published:** 2017-01-26

**Authors:** Andrew L. Rhyne, Michael F. Tlusty, Joseph T. Szczebak, Robert J. Holmberg

**Affiliations:** 1 Biology and Marine Biology, Roger Williams University, Bristol, RI, United States; 2 Anderson Cabot Center for Ocean Life, New England Aquarium, Boston, MA, United States; 3 School for the Environment, University of Massachusetts Boston, Boston, MA, United States; 4 Center for Economic and Environmental Development, Roger Williams University, Bristol, RI, United States

**Keywords:** Marine aquarium trade, Wildlife trade, Coral reef, Data visualization

## Abstract

The trade of live marine animals for home and public aquaria has grown into a major global industry. Millions of marine fishes and invertebrates are removed from coral reefs and associated habitats each year. The majority are imported into the United States, with the remainder sent to Europe, Japan, and a handful of other countries. Despite the recent growth and diversification of the aquarium trade, to date, data collection is not mandatory, and hence comprehensive information on species volume and diversity is lacking. This lack of information makes it impossible to study trade pathways. Without species-specific volume and diversity data, it is unclear how importing and exporting governments can oversee this industry effectively or how sustainability should be encouraged. To expand our knowledge and understanding of the trade, and to effectively communicate this new understanding, we introduce the publically-available Marine Aquarium Biodiversity and Trade Flow online database (https://www.aquariumtradedata.org/). This tool was created to communicate the volume and diversity of marine fishes and/or invertebrates imported into the US over three complete years (2008, 2009, and 2011) and three partial years (2000, 2004, 2005). To create this tool, invoices pertaining to shipments of live marine fishes and invertebrates were scanned and analyzed for species name, species quantities, country of origin, port of entry, and city of import destination. Here we focus on the analysis of the later three years of data and also produce an estimate for the entirety of 2000, 2004, and 2005. The three-year aggregate totals (2008, 2009, 2011) indicate that just under 2,300 fish and 725 invertebrate species were imported into the US cumulatively, although just under 1,800 fish and 550 invertebrate species were traded annually. Overall, the total number of live marine animals decreased between 2008 and 2011. In 2008, 2009, and 2011, the total number of individual fish (8.2, 7.3, and 6.9 million individuals) and invertebrates (4.2, 3.7, and 3.6 million individuals) assessed by analyzing the invoice data are roughly 60% of the total volumes recorded through the Law Enforcement Management Information System (LEMIS) dataset. Using these complete years, we back-calculated the number of individuals of both fishes and invertebrates imported in 2000, 2004, and 2005. These estimates (9.3, 10.8, and 11.2 million individual fish per year) were consistent with the three years of complete data. We also use these data to understand the global trade in two species (Banggai cardinalfish, *Pterapogon kauderni*, and orange clownfish, *Amphiprion ocellaris* / *percula*) recently considered for Endangered Species Act listing. Aquariumtradedata.org can help create more effective management plans for the traded species, and ideally could be implemented at key trade ports to better assess the global trade of aquatic wildlife.

## Introduction

The live aquarium fish trade faces a multitude of potential threats, including incidences of reduced biodiversity from over-extraction, habitat destruction in some source countries (Francis-Floyd & Klinger, 2003; Gopakumar & Ignatius, 2006), and negative impacts of species invasions in the US and elsewhere (Chucholl, 2013; García-Berthou, 2007; Holmberg et al., 2015; Padilla & Williams, 2004). Despite these threats, the aquarium trade can be a unique and significant positive force in reef-side communities (Rhyne, Tlusty & Kaufman, 2014). Benefits include but are not limited to saving threatened species from the brink of extinction through the development of captive breeding programs (Tlusty, 2002) and catalyzing habitat preservation through sustainable supply-side practices (Firchau, Dowd & Tlusty, 2013). These sustainable practices include stewardship, mechanisms for sustainable livelihoods via poverty alleviation, and the protection of threatened ecosystems (Rhyne, Tlusty & Kaufman, 2014). Finally, consumer education of aquarium trade sustainability can promote widespread public appreciation for the world’s aquatic ecosystems, with the ultimate goal of minimizing negative impacts of this trade (Tlusty et al., 2013). While a proactive stance can transform a large consumer base into a powerful agent for biodiversity conservation, increased sustainability, and human well-being, inaction will likely amplify the deleterious threats currently faced by the trade. Currently, the lack of oversight leading to a poor concept of trade volume and subsequent regulatory inefficiency has greatly hampered the development of a sustainable industry. Increasing the sustainability of the marine aquarium animal industry should be considered a primary initiative for the entire aquarium industry transport chain (Tlusty et al., 2013). Increasing the sustainability of the transport chain of marine aquarium animals is achieved through a more thorough understanding of the magnitude of the trade (Fujita et al., 2014), which begins by assessing the scale of imports into the US (the primary destination) (Rhyne et al., 2012). Once the annual volume of US imports is gauged, other relevant issues that lead to environmental and economic benefits can then be tackled, including animal quality and shipping survival.

There is no clear picture of the number of live marine fish and invertebrate species or individuals involved in the aquarium trade, primarily due to insufficient global tracking of the import and export of these animals (Bruckner, 2001; Fujita et al., 2014; Green, 2003; Lunn & Moreau, 2004; Tissot et al., 2010; Wabnitz et al., 2003). Multiple sources of data have been used to monitor this trade (Green, 2003; Smith et al., 2008; Wabnitz et al., 2003; Wood, 2001). However, not all of these data systems are sufficient for, or were even intended for, monitoring the aquarium trade. For example, compulsory data are maintained under federal mandates for species listed by the Convention on International Trade in Endangered Species of Wild Fauna and Flora (CITES). However, previous studies found that CITES records were inaccurate, incomplete, or insufficient (Bickford et al., 2011; Blundell & Mascia, 2005; Rhyne et al., 2012). Furthermore, CITES-listed species (namely stony corals, giant clams, and seahorses) account for only a fraction of the total trade in live aquatic animals. Only a handful of studies (Rhyne et al., 2012; Smith et al., 2009; Smith et al., 2008) have attempted to quantify the movement of non-CITES-listed aquarium species from source to market. The Global Marine Aquarium Database (GMAD) encouraged companies to provide data on the marine aquarium trade (Green, 2003) and, until now, GMAD was the only source for aquarium trade data recorded at a species-specific level. While GMAD contains trade data from 1988 to 2003, some years and countries (e.g., Haiti) are missing, and values reported in GMAD were found to be drastically fewer than other estimates of trade data (Murray et al., 2012). Furthermore, the aquarium trade has been transformed by new technologies and husbandry breakthroughs since 2003 (Rhyne & Tlusty, 2012) which may dilute the relevance of this data set. In addition to CITES and GMAD, the Law Enforcement Management Information System (LEMIS) database has been used to better understand the aquarium trade. The United States Fish and Wildlife Service (USFWS) inspects wildlife shipments and, through LEMIS, maintains species-specific data in the case of transporting CITES-listed species per CITES requirements. However, non-CITES-listed species are recorded with general codes (e.g., marine aquarium tropical fish, regardless of species, are coded MATF). Recording data in this generalized manner eliminates specific information regarding the diversity and volumes of species traded (Smith et al., 2009). While the need for accurate accounts of aquarium trade flow continually increases, the current monitoring methods remain static (Bickford et al., 2011). The lack of specific data systems for recording all species exported and imported for the marine aquarium trade raises two main concerns: (1) because of the lack of trade data, it is unclear how importing and exporting governments can monitor this industry effectively; (2) it is also unclear how sustainability should be encouraged given the paucity of data.

To date, Balboa (2003), Wabnitz et al. (2003) and Rhyne et al. (2012), have catalogued species-specific information provided on trade invoices. Rhyne et al. (2012) further compared invoice information to associated shipment declarations. It was this effort that led to the development of the Marine Aquarium Biodiversity and Trade Flow online database (https://www.aquariumtradedata.org/), a public portal offering anonymized live marine animal trade data collected through trade invoices. Here we describe three years (2008, 2009, 2011) of fish and invertebrate invoice-based data from US imports that were analyzed for country of origin, port of entry, and quantity of species and individuals associated with each port. We also relate the findings from these three years of complete trade data to previously existing invoice-based trade data from the LEMIS database. Specifically, Rhyne et al. (2012) described one contiguous year of import data, based on a 12-month period from June 2004 (seven months) until May 2005 (five months), and Balboa (2003) described one month (October) of data from 2000. To address the missing months of data from these years and to increase the scope of the dataset, we modeled data for the missing months based on the monthly volume patterns analyzed from the three complete years of data available (2008, 2009, 2011). This work provides an enlarged snapshot of the volume, biodiversity, and trade pathways for live marine aquarium fish and invertebrate species beyond the information provided by other reporting systems (Wabnitz et al., 2003). Further, this work demonstrates that LEMIS, while well-designed for import/export compliance and management of USFWS staffing needs, is not designed to monitor the species-level activity of the live marine aquarium trade. Finally, we present two case studies (the Banggai cardinalfish, *Pterapogon kauderni*, and the common/orange clownfish, *Amphiprion ocellaris/percula*) to demonstrate the use of these data for better understanding the trade in marine species: a first step toward advancing industry sustainability.

## Methods

The goal of this project was to evaluate the number of live marine aquarium species imported into the US, and to create a trade path analysis of the diversity of animals involved in the trade. The methods used to analyze trade invoices were described by Rhyne et al. (2012) and are briefly summarized here. We reviewed all USFWS shipment declarations and the attached commercial invoices coded as Marine Aquarium Tropical Fish (MATF) for 2008, 2009 and 2011 as indicated in the LEMIS database. While about 22,000 invoices were marked as containing MATF in the LEMIS database, we received approximately 20,000 shipment declarations and their attached invoices. CITES data are specially coded on import declarations, and thus are not integrated into this database.

**Figure 1:**
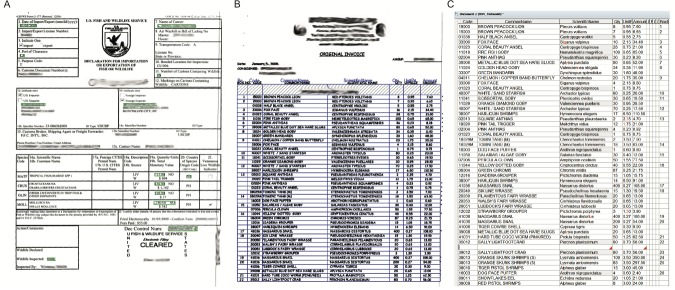
FlexiCapture 9.0 verification screen used to capture shipping data for the www.aquariumtradedata.org database. (A) Shipping declaration; (B) shipping invoice; (C) invoice table produced from Optimal Character Recognition (OCR) software. Note: grey shaded cells indicate autocorrected fields and red flags within cells indicate errors for user to correct manually.

Invoices were considered a true statement of shipping contents, as we were not able to assess the veracity of the information contained on the invoice. Shipment information (date, port of origin, and destination port) was collected from the declaration page, and species and quantity information was tabulated from the associated invoices and catalogued into a database. Within the trade of live marine organisms, re-export information is not recorded, and thus could not be assessed here. Both manual entry and automated optical character recognition (OCR) software (ABBYY FlexiCapture 9.0) customized for wildlife shipments (Fig. 1) were utilized to retrieve the above information from these documents. The input method varied with invoice quality and length. Manual entry was utilized when invoices were of poor quality (blurry, speckled, darkened, fonts less than six point, handwritten) or brief (less than 1/2 page), whereas all others were read using the OCR software. Once all necessary data were captured, species names were verified using World Register of Marine Species (WoRMS Editorial Board, 2015), FishBase (Froese & Pauly, 2015), and the primary literature (Appeltans et al., 2011; Froese & Pauly, 2011). We corrected species information only when species names were misspelled, listed under a junior synonym, or listed by only a common name. Species were identified to the greatest taxonomic detail available. Occasionally, genera were listed without species (e.g., ‘*Chrysiptera* sp.’), or the species was otherwise ambiguous (e.g., ‘hybrid *Acanthurus* tang’). In these cases the genus (and all higher-level taxonomic information) would be recorded, and the species would be recorded as unknown. Genus and species were recorded as unknown when listed only by an ambiguous common name (e.g., ‘colorful damsel,’ ‘unknown damsel,’ ‘assorted damsels’).

To determine if the volume of captive-bred *P. kauderni* imported into the US has increased in recent years, we reviewed invoice data from Los Angeles-based importer Quality Marine for two additional recent years of imports. Quality Marine sourced fish from Thailand, and these were known to have been captive-bred given that Thailand lies outside the native range of *P. kauderni*. At our request, all shipments of MATF from Thailand to Quality Marine over the period of March 2012 to July 2014 were supplied and reviewed.

In accordance with Rhyne et al. (2012), this report focused on major geographic trade flows, the frequency of invoice detail to species-level, and how invoice data compared to LEMIS data. Invoice data for both fishes and invertebrates were retrieved concurrently. To help organize and visualize the trade data, a publically accessible representation of the trade data was created: the Marine Aquarium Biodiversity and Trade Flow online database (https://www.aquariumtradedata.org/). This web-based graphical user interface, powered by the open source JavaScript library D3 (http://d3js.org/), is data-rich, visually appealing, and allows users to query more than 28,000 invoices containing over 2.7 million lines of invoice data from 2000, 2004, 2005, 2008, 2009, and 2011.

To back-calculate the estimated total annual imports of marine animals during years with incomplete data sets (years 2000, 2004, 2005), we first determined the proportion of individuals imported during the time interval (one month for 2000, seven months for 2004, and five months for 2005) based on the three years for which we had a complete 12-month dataset (2008, 2009, and 2011). For these three years, there was variation between months, but the intra-month variation was less than that of the inter-month variation, suggesting that monthly import volumes were proportionally consistent. This proportion was then used to calculate the number of individuals for the unknown months using the following relation:

\begin{eqnarray*}(n/\bar {Pr})-n \end{eqnarray*}

where *n* is the known number of imports per year for 1 (2000), 5 (2004) or 7 (2005) months, and *Pr* is the average proportion of known imports from corresponding months from 2008, 2009 and 2011. This estimated number of animals was then allocated across the unknown months proportionally for 2000, 2004 and 2005. This method appears robust given the consistency of the most voluminous species across years (see results). We also generated estimates for the source countries and ports of entry. The database user can select the type of data to view, and within the selection tool, can select to view actual data or whether estimates should be included. Even though these numbers are estimates, we present them as numbers so that they coalesce with the complete database.

## Results

**Figure 2:**
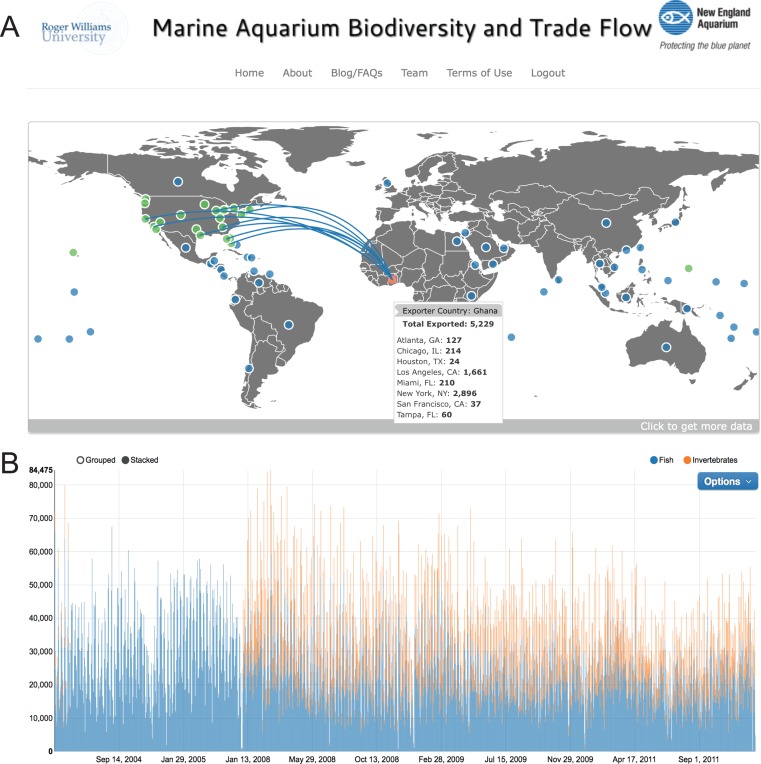
Main dashboard page of www.aquariumtradedata.org. (A) Interactive trade flow map depicting exporting countries (blue circles) and ports of entry in the US (green circles); (B) Timeline chart of fishes and invertebrates imported into the US based on user-selected dates.

**Figure 3:**
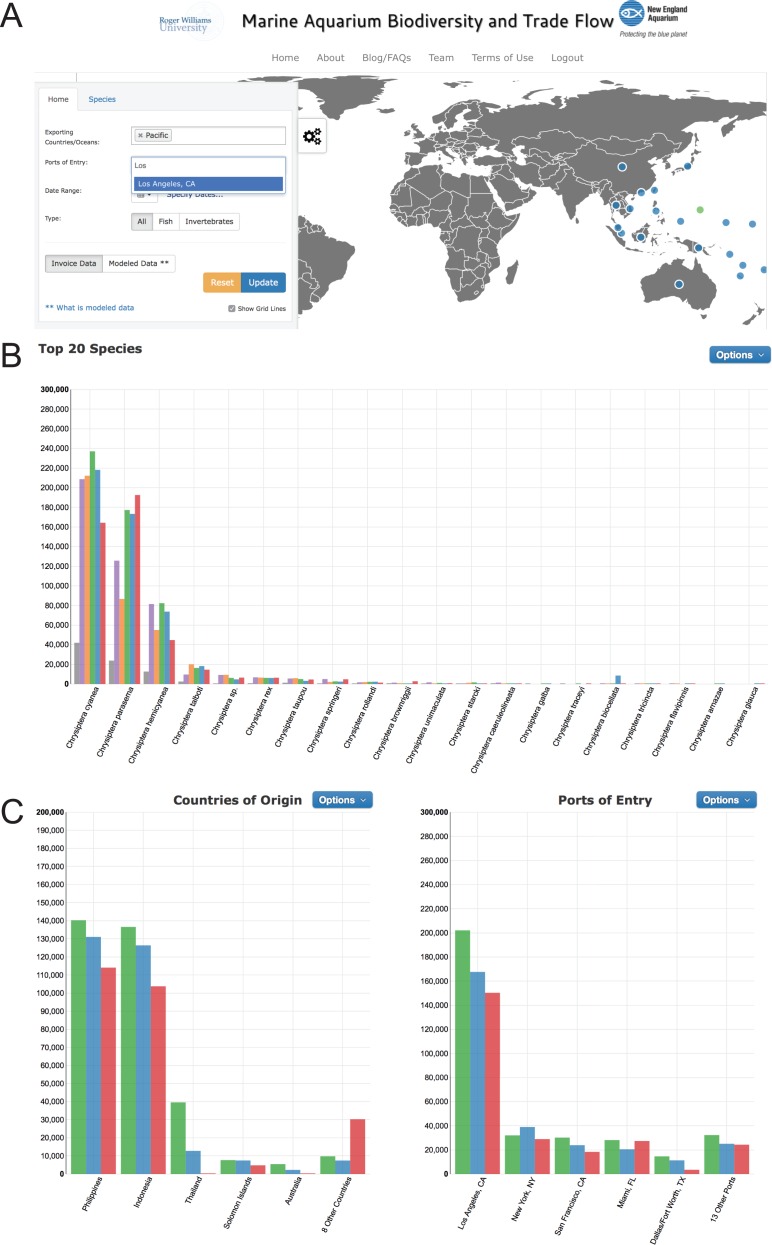
Drop down menus for user-generated queries in www.aquariumtradedata.org. (A) Main tab with Exporting Countries/Ocean and Ports of Entry inputs; (B) Species tab with taxa selection displaying “Top 20 Species” chart; (C) “Countries of Origin” and “Ports of Entry” charts generated by the query.

**Figure 4:**
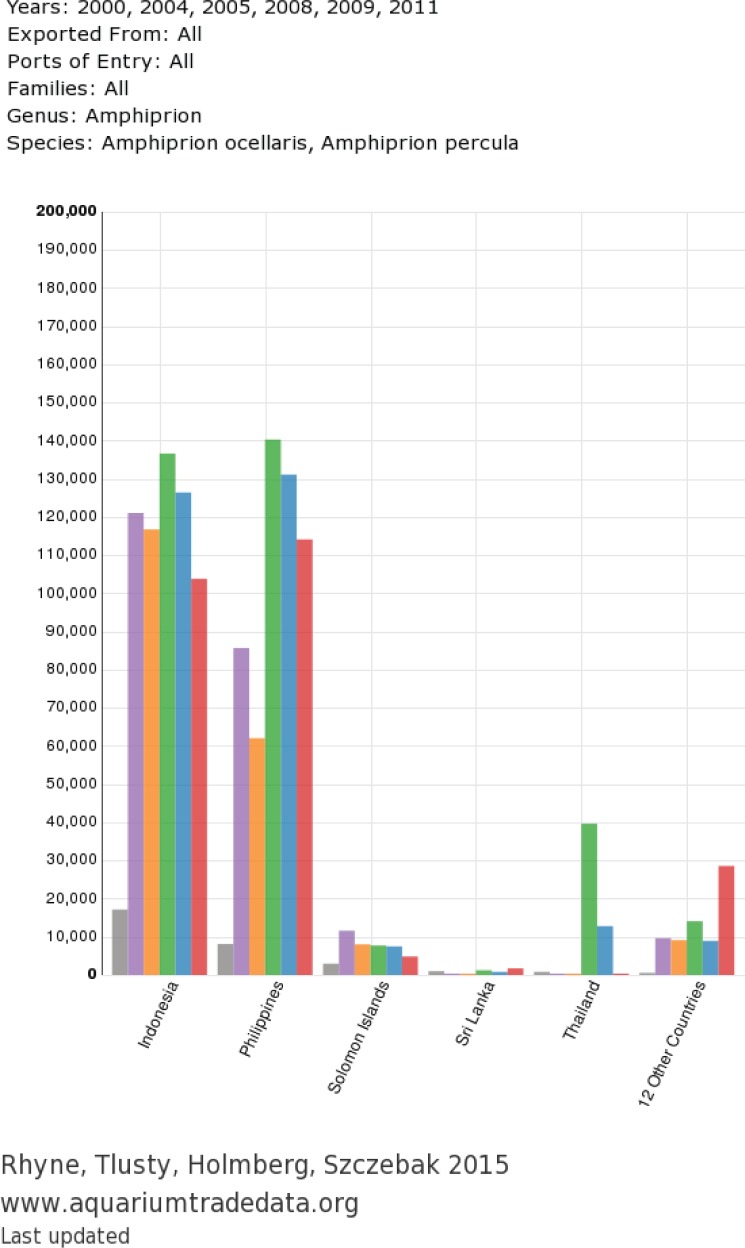
Exported chart from user-generated query: countries of origin for *A. percula* and *A. ocellaris* in www.aquariumtradedata.org. Export header includes query details and export footer includes data attributes and date of last database update.

All data presented in this report are available at the Marine Aquarium Biodiversity and Trade Flow online database (https://www.aquariumtradedata.org/). This site was developed to allow users to generate database queries using dropdown menus. On the “Home” tab, initial queries can be filtered through large-scale source areas such as ocean basins or countries of origin for a defined time period (Fig. 2). Following user selections, the software compiles detailed information in the form of maps, timeline charts, and other data charts that allow users to access data at a depth uncommon in user interfaces for the wildlife or seafood trades. On further analysis, it is possible, using the “Species” tab, to query a single taxonomic family, genus, or species for one or more countries and/or ports of entry. The user-friendly dropdown menus are tree-based and progressive. Figure 3 demonstrates successive screens where the user has successively selected the family Pomacentridae, the genus *Amphiprion*, and the species complex *Amphiprion percula*/*ocellaris*. The dashboard displays (A) a distribution map depicting the relative geographic importance using proportionally-sized red dots, and (B, C) two graphs displaying export country- and port of entry-specific volumes for the selected query. To enhance the utility of the website and promote the dissemination of the data, the user can download charts and graphs of data queries. Users can also share these charts directly to Facebook and Twitter (Fig. 4). Further, to ensure the data within the invoice-based database is an accurate representation of the trade, users can report possible errors in data or features on the website. If users find species that are likely incorrect in distribution or taxa, we can examine the invoice record, verify its contents, and update the database if needed. This system also logs how users interact with the database, which provides feedback on the number and types of queries users generated.

### General trends

In 2008, a total of 8,299,467 individual fishes (97.4% identified to species-level) representing 1,788 species were imported into the US. The total number of fishes imported decreased to 7,102,246 in 2009 and to 6,892,960 in 2011. However, the number of species imported increased to 1,798 by 2011. While no more than 1,800 species were imported in a single year, 2,278 unique species were imported across the three-year span (Table 1).

A similar trend in the overall trade was observed for non-CITES-listed invertebrates during this time period, although the invertebrate data were less voluminous and specious compared to the fish data. A total of 4.3 million invertebrates representing 545 species were imported into the US in 2008. The total number of invertebrates imported decreased to about 3.7 million in 2009 and 2011 (Table 2). A total of 724 species were imported over the three-year span. Compared to fishes, relatively fewer invertebrates were identified to species-level (72.9%).

### Export countries

**Figure 5:**
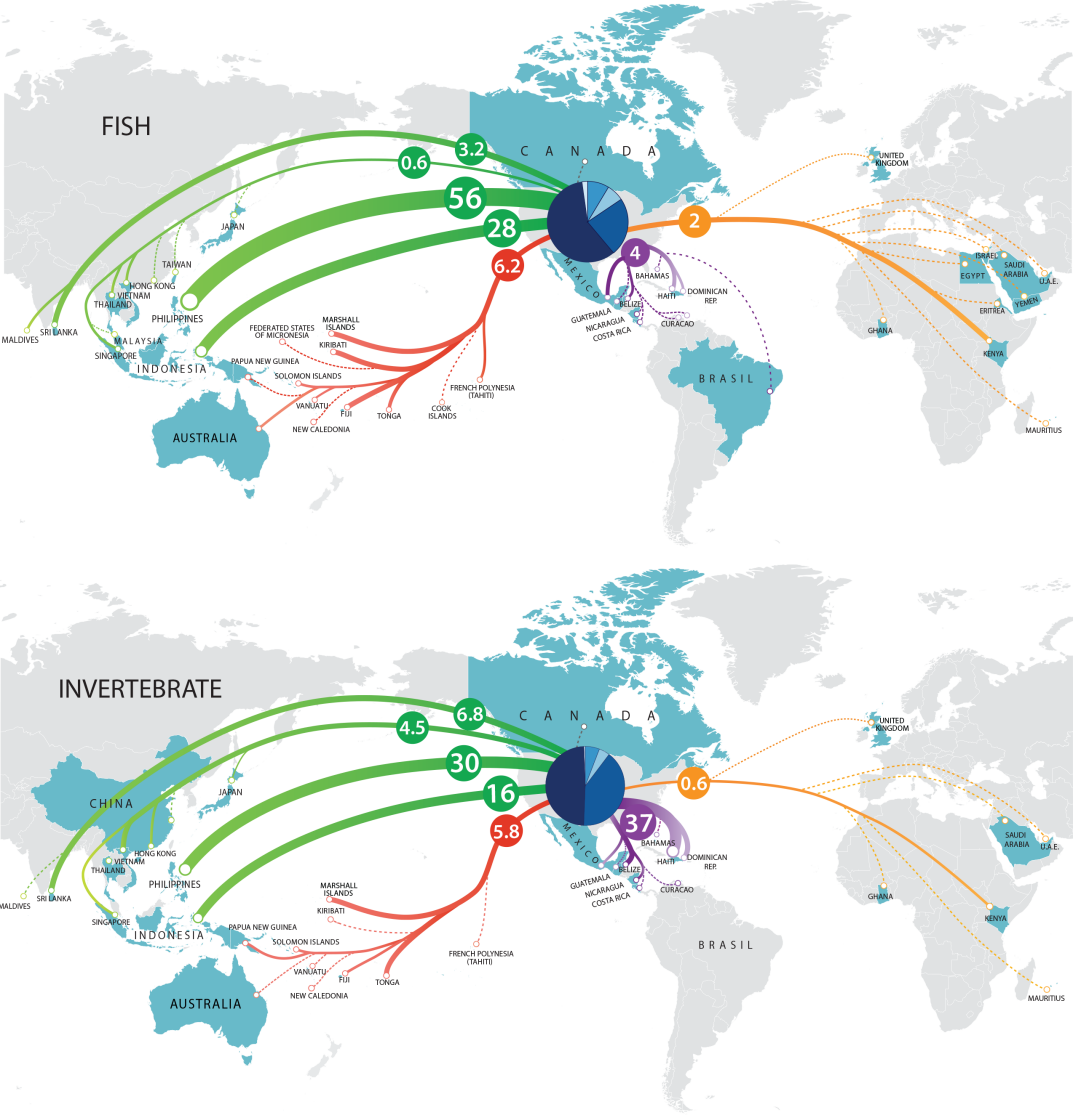
Trade flow of marine aquarium fishes and invertebrates from source nations into the US during 2008, 2009 and 2011. Numbers within circles denote percent contribution to total imports. Pie chart within US represents Ports of Entry (with the Midwest starting at 0 degrees, and clockwise, NE, SE, SW and NW).

Forty-five countries in total exported marine fishes to the US during the three years (Table 1), with 41, 37, and 36 countries noted in 2008, 2009, and 2011, respectively. The Philippines exported 56% of the cumulative total volume (12.7 million fishes, Fig. 5). The overall volume of fishes traded decreased by 17% between 2008 and 2011, with commensurate export decreases from the Philippines and Indonesia. Third-ranked Sri Lanka exported consistently across the three years. Exports from fourth-ranked Haiti decreased by nearly 50% between 2008 and 2011, likely due to earthquake activity in 2010.

**Table 1:** Countries that exported live aquarium fishes to the US. Data include the number of identifiable species, number of species exported in quantities greater than 1,000 individuals (>1,000 (No.)), number of individuals exported across species, and the proportion of individuals identifiable to species-level (Known (%)) by country and year.

Export Country	2008				2009				2011				2008–2011			
	Species		Individuals		Species		Individuals		Species		Individuals		Species		Individuals	
	Total (No.)	>1,000 (No.)	Total (No.)	Known (%)	Total (No.)	>1,000 (No.)	Total (No.)	Known (%)	Total (No.)	>1,000 (No.)	Total (No.)	Known (%)	Total (No.)	>1,000 (No.)	Total (No.)	Known (%)
Australia	115	3	12,877	100.0	162	2	8,773	100.0	199	1	9,573	99.6	298	6	31,224	99.9
Belize	49	4	9,472	99.9	45	3	8,846	99.4	39	4	14,976	99.6	63	6	33,351	99.6
Brazil	71	3	8,742	99.4	61	0	3,349	98.5	45	0	2,005	99.5	87	2	14,147	99.2
Canada	2	0	52	100.0	2	0	3	100.0	1	0	26	42.3	5	0	81	81.5
Cook Islands	23	2	4,763	100.0	27	2	3,317	100.0	–	–	–	–	34	2	8,080	100
Costa Rica	22	2	7,903	100.0	46	0	3,538	100.0	28	2	6,139	88.1	53	4	17,580	95.8
Curaçao	15	0	529	100.0	26	0	1,383	100.0	34	1	3,367	99.7	47	1	5,279	99.8
Dominican Republic	26	3	22,121	86.3	19	4	28,944	77.2	48	8	34,272	96.7	52	8	93,872	86.5
Egypt	–	–	–	–	–	–	–	–	20	0	953	92.1	20	0	953	92.1
Eritrea	52	2	9,506	99.6	44	0	3,986	99.3	–	–	–	–	62	2	13,519	99.5
Fed. States of Micronesia	–	–	–	–	–	–	–	–	131	0	5,550	97.4	131	0	5,550	97.4
Fiji	187	28	115,520	98.9	228	19	88,289	97.8	311	25	156,680	97.6	363	44	362,444	98.1
French Polynesia (Tahiti)	106	6	42,846	99.9	101	3	30,187	99.5	73	3	29,011	99	144	7	102,182	99.5
Ghana	19	0	509	99.8	19	0	686	96.1	22	0	708	95.5	33	0	1,931	96.8
Guatemala	3	0	1,055	100	3	0	343	100.0	–	–	–	–	3	0	1,398	100.0
Haiti	99	23	240,552	97.7	114	23	215,909	97.6	89	19	126,799	99	133	30	588,516	97.9
Hong Kong	9	0	262	99.2	4	0	5	100	1	0	16,510	0.3	14	0	16,777	1.9
Indonesia	973	214	2,402,733	97.2	1,009	186	1,998,195	96.9	992	181	1,867,946	97.3	1,284	234	6,331,781	97.2
Israel	–	–	–	–	7	0	666	100.0	10	2	21,985	100.0	10	0	22,651	100.0
Japan	44	0	1,133	100.0	92	0	1,137	100.0	62	0	569	92.4	132	0	2,839	98.5
Kenya	173	27	144,211	97.7	210	24	139,129	97.8	186	21	101,910	99	249	39	388,376	98.1
Kiribati	67	6	122,971	99.1	52	7	78,812	98.2	72	6	105,679	97.6	103	8	308,889	98.4
Malaysia	11	0	622	99.8	–	–	–	–	1	0	13	100.0	12	0	635	99.8
Mauritius	63	0	823	93.4	–	–	–	–	41	0	680	98.2	81	0	1,503	95.6
Mexico	90	1	5,174	99.4	40	2	12,688	96.2	62	3	15,135	98.6	118	5	33,504	97.8
Netherlands Antilles	9	0	319	100.0	–	–	–	–	–	–	–	–	9	0	319	100.0
New Caledonia	2	0	84	100.0	2	0	75	100.0	17	0	387	99.7	17	0	546	99.8
Nicaragua	72	2	8,986	98.0	–	–	–	–	31	0	1,847	93.2	83	1	10,833	97.2
Papua New Guinea	132	2	6,816	99.8	111	2	8,313	98.6	–	–	–	–	176	2	15,243	99.1
Philippines	980	255	4,694,961	97.5	1,053	248	4,024,693	97.3	1,016	258	3,901,058	97.3	1,320	315	12,732,212	97.4
Rep. of Maldives	141	5	24,574	96.4	109	4	22,093	98.9	67	11	34,360	100	174	19	81,275	98.6
Rep. of the Marshall Isl	96	6	37,972	94.0	138	9	115,686	75.1	139	13	142,068	78.7	227	19	334,174	78.8
Saudi Arabia	16	0	326	100.0	4	0	19	100.0	–	–	–	–	20	0	345	100.0
Singapore	36	1	2,606	100.0	14	1	2,520	99.8	42	4	13,949	99.5	71	3	19,081	99.6
Solomon Islands	134	8	47,262	96.5	133	6	34,773	94.9	138	10	41,673	92.5	180	15	125,588	94.7
Sri Lanka	419	30	202,632	98.0	468	34	217,116	97.1	461	28	212,407	96.7	633	57	638,606	97.2
Taiwan	33	0	1,511	98.1	29	0	897	85.3	26	1	2,444	100.0	63	0	5,007	96.3
Thailand	10	3	39,887	100.0	3	1	8,310	100.0	–	–	–	–	10	0	48,197	100.0
The Bahamas	85	0	951	100.0	45	0	432	99.8	8	0	297	100.0	98	0	1,681	99.9
Tonga	207	6	27,857	89.5	92	2	8,047	92.3	82	0	2,676	91	227	8	39,253	90.2
United Arab Emirates	–	–	–	–	–	–	–	–	7	0	77	85.7	7	0	77	85.7
United Kingdom	32	1	3,710	98.6	–	–	–	–	–	–	–	–	32	0	3,710	98.6
Vanuatu	190	3	19,704	97.2	123	1	12,671	96.8	183	0	14,405	94.1	240	11	47,195	96.2
Vietnam	146	1	14,593	99.8	112	1	6,545	99.1	102	0	4,826	99.5	183	6	26,022	99.6
Yemen	10	3	10,340	100	14	3	11,871	100.0	–	–	–	–	16	3	22,211	100.0
**Total**	**1,788**	**443**	**8,299,467**	**97.4**	**1,780**	**411**	**7,102,246**	**96.7**	**1,798**	**413**	**6,892,960**	**96.7**	**2,278**	**518**	**22,538,637**	**97.0**

**Table 2:** Countries that exported live aquarium invertebrates to the US. Data include the number of identifiable species, number of species exported in quantities greater than 1,000 individuals (>1,000 (No.)), number of individuals exported across species, and the proportion of individuals identifiable to the species-level (Known (%)) by country and year.

Export Country	2008				2009				2011				2008–2011			
	Species		Individuals		Species		Individuals		Species		Individuals		Species		Individuals	
	Total (No.)	>1,000 (No.)	Total (No.)	Known (%)	Total (No.)	>1,000 (No.)	Total (No.)	Known (%)	Total (No.)	>1,000 (No.)	Total (No.)	Known (%)	Total (No.)	>1,000 (No.)	Total (No.)	Known (%)
Australia	3	0	231	37.2	16	0	1,881	99.8	34	0	1,020	90.6	43	1	3,132	92.2
Belize	8	2	49,515	56.1	7	3	83,922	57.3	12	6	292,176	58.2	17	7	425,613	57.8
Brazil	–	–	–	–	–	–	1	0.0	–	–	–	–	–	–	1	0.0
Canada	1	0	2	100.0	–	–	–	–	–	–	28	0.0	1	0	30	6.7
China	3	0	1,260	100.0	–	–	–	–	–	–	–	–	3	0	1,260	100
Costa Rica	–	–	–	–	3	0	64	100.0	–	–	–	–	3	0	64	100
Curaçao	–	–	–	–	1	0	15	100.0	4	0	911	89.1	5	0	926	89.3
Dominican Republic	6	2	93,781	99.9	5	2	133,056	100.0	18	4	107,103	96.3	19	4	333,940	98.8
Federated States of Micronesia	–	–	–	–	–	–	–	–	–	–	1	0.0	–	–	1	0.0
Fiji	6	2	52,228	15.4	9	1	25,502	20.5	23	3	28,462	22.4	29	4	106,192	18.5
French Polynesia (Tahiti)	–	–	766	0.0	3	0.0	48	47.9	–	–	–	–	3	0	814	2.8
Ghana	3	0	2,395	12.2	3	0	135	100.0	3	0	768	53.5	5	0	3,298	25.4
Guatemala	–	–	3,000	0.0	–	–	–	–	–	–	–	–	–	–	3,000	0.0
Haiti	55	24	1,409,841	92.5	63	24	1,011,683	93.3	47	26	676,134	94.4	79	34	3,097,658	93.2
Hong Kong	1	1	1,520	100.0	–	–	–	–	1	1	23,255	100.0	1	1	24,775	100.0
Indonesia	323	57	709,736	61.2	317	51	610,264	64.9	301	48	575,657	68.6	413	90	1,895,657	64.6
Japan	8	0	425	76.5	17	0	556	64	12	0	168	91.1	25	0	1,149	72.6
Kenya	18	2	13,955	57.0	17	2	44,426	26.1	22	1	14,750	53.7	30	6	73,131	37.6
Kiribati	–	–	6	0.0	–	–	18	0.0	1	0	80	15	1	0	104	11.5
Mauritius	1	0	198	100.0	–	–	–	–	–	–	–	–	1	0	198	100.0
Mexico	24	1	1,429	99.4	8	2	4,035	97.6	17	2	17,678	53.3	38	5	23,142	63.9
New Caledonia	–	–	–	–	–	–	–	–		–	4	0.0	–	–	4	0.0
Nicaragua	30	8	58,918	83.8	–	–	–	–	19	3	31,052	74.5	41	11	89,970	80.6
Papua New Guinea	23	0	2,323	90.5	21	2	6,336	83.9		–	–	–	34	3	8,659	85.6
Philippines	259	65	1,111,002	71.5	294	67	1,154,255	65.6	284	75	1,380,014	68.2	395	118	3,645,271	68.4
Rep. of Maldives	3	0	95	21.1	2	0	686	2.6		–	890	0.0	4	0	1,671	2.3
Rep. of the Marshall Islands	3	2	47,362	100.0	7	5	200,088	42.1	24	3	39,588	68.8	29	6	287,038	55.4
Saudi Arabia	–	–	–	–	–	–	5	0.0		–	–	–	–	–	5	0.0
Singapore	15	0	2,654	45.9	11	0	2,063	64.7	11	1	7,017	56.7	21	2	11,734	55.7
Solomon Islands	17	2	12,521	51.4	10	1	4,084	67.4	8	2	16,753	43.7	21	3	33,358	49.5
Sri Lanka	63	11	251,373	90.2	60	9	309,053	91.9	54	11	261,004	88.1	87	17	821,430	90.2
Thailand	1	0	250	100.0	–	–	–	–	–	–	–	–	1	0	250	100.0
The Bahamas	9	0	92	97.8	6	0	28	78.6	–	–	–	–	13	0	120	93.3
Tonga	18	3	135,089	65.6	8	2	31,214	61.0	8	1	81,918	52.6	23	3	248,221	60.7
United Arab Emirates	–	–	–	–	–	–	–	–	–	–	2	0.0	–	–	2	0.0
United Kingdom	–	–	–	–	–	–	–	–	2	0	4	100.0	2	0	4	100.0
Vanuatu	8	0	672	99.9	4	0	96	97.9	5	0	132	79.5	11	0	900	96.7
Vietnam	25	6	293,733	8.1	23	5	108,699	27.2	24	4	106,411	12.2	38	8	508,843	13.1
**Total**	**545**	**137**	**4,256,372**	**73.4**	**537**	**126**	**3,732,213**	**73.1**	**535**	**138**	**3,662,980**	**72.2**	**724**	**220**	**11,651,565**	**72.9**

The US imported marine invertebrates from a total of 38 countries during the three years (Fig. 5, Table 2), although only 27 (2008, 2009) or 28 (2011) countries were noted per year. The number of individuals exported per year decreased 14% between 2008 and 2011, a rate similar to that of fishes. The countries exporting the greatest volume over the three years were the Philippines (3.6 million invertebrates) and Haiti (3.1 million invertebrates). The number of individual invertebrates exported from the Philippines increased by 24% between 2008 and 2011. This was likely a response to the decrease in volume from Haiti (52% decline from 2008 to 2011). Third-ranked Indonesia (1.8 million invertebrates) exported a consistent volume across the three years. Even though Indonesia ranked third in volume, it exported the most species (413) during the three years. The Philippines (395) and Sri Lanka (87) were second and third respectively in terms of the number of species exported to the US.

**Table 3:** Top 20 live aquarium fish and invertebrate species imported into the US by year. Data include proportion of import volume (individuals as % Total) and number of export countries (Countries (No.)) for each.

Rank	2008			2009			2011		
	Species	% Total	Countries (No.)	Species	% Total	Countries (No.)	Species	% Total	Countries (No.)
**Fishes**									
1	*Chromis viridis*	10.2%	13	*Chromis viridis*	10.5%	16	*Chromis viridis*	11.6%	13
2	*Chrysiptera cyanea*	5.6%	8	*Chrysiptera cyanea*	5.0%	8	*Chrysiptera parasema*	4.7%	6
3	*Dascyllus trimaculatus*	5.0%	11	*Dascyllus trimaculatus*	4.4%	12	*Chrysiptera cyanea*	4.4%	7
4	*Dascyllus aruanus*	3.7%	9	*Chrysiptera parasema*	3.6%	4	*Dascyllus trimaculatus*	3.7%	10
5	*Chrysiptera parasema*	3.5%	3	*Dascyllus aruanus*	3.3%	9	*Dascyllus aruanus*	3.6%	8
6	*Amphiprion ocellaris*	3.2%	10	*Amphiprion ocellaris*	3.0%	10	*Nemateleotris magnifica*	3.0%	8
7	*Nemateleotris magnifica*	2.7%	12	*Nemateleotris magnifica*	2.4%	12	*Amphiprion ocellaris*	3.0%	10
8	*Chrysiptera hemicyanea*	2.6%	2	*Chrysiptera hemicyanea*	2.4%	3	*Pterapogon kauderni*	1.9%	5
9	*Dascyllus melanurus*	1.8%	3	*Dascyllus melanurus*	2.0%	6	*Centropyge loricula*	1.9%	9
10	*Pterapogon kauderni*	1.8%	3	*Paracanthurus hepatus*	1.7%	14	*Pseudocheilinus hexataenia*	1.6%	9
11	*Pseudocheilinus hexataenia*	1.4%	13	*Pterapogon kauderni*	1.7%	4	*Dascyllus melanurus*	1.6%	3
12	*Paracanthurus hepatus*	1.3%	16	*Centropyge loricula*	1.6%	7	*Sphaeramia nematoptera*	1.5%	7
13	*Synchiropus splendidus*	1.3%	3	*Pseudocheilinus hexataenia*	1.6%	12	*Chrysiptera hemicyanea*	1.5%	3
14	*Centropyge loricula*	1.3%	8	*Valenciennea puellaris*	1.4%	10	*Synchiropus splendidus*	1.5%	5
15	*Labroides dimidiatus*	1.3%	13	*Synchiropus splendidus*	1.4%	5	*Valenciennea puellaris*	1.4%	7
16	*Salarias fasciatus*	1.3%	8	*Gramma loreto*	1.3%	6	*Paracanthurus hepatus*	1.3%	15
17	*Gramma loreto*	1.2%	6	*Salarias fasciatus*	1.3%	10	*Salarias fasciatus*	1.2%	11
18	*Valenciennea puellaris*	1.1%	9	*Sphaeramia nematoptera*	1.3%	6	*Centropyge bispinosa*	1.2%	14
19	*Centropyge bispinosa*	1.1%	12	*Labroides dimidiatus*	1.1%	13	*Labroides dimidiatus*	1.1%	11
20	*Sphaeramia nematoptera*	1.0%	7	*Centropyge bispinosa*	1.1%	12	*Valenciennea strigata*	0.9%	10
**Invertebrates**									
1	*Paguristes cadenati*	22.0%	3	*Paguristes cadenati*	20.9%	2	*Paguristes cadenati*	14.0%	4
2	*Lysmata amboinensis*	10.2%	6	*Lysmata amboinensis*	13.5%	8	*Lysmata amboinensis*	12.3%	8
3	*Clibanarius tricolor*	8.0%	1	*Clibanarius tricolor*	5.7%	2	*Mithraculus sculptus*	7.3%	3
4	*Mithraculus sculptus*	5.1%	3	*Mithraculus sculptus*	4.2%	2	*Trochus maculatus*	4.6%	3
5	*Stenopus hispidus*	2.9%	11	*Lysmata debelius*	3.0%	5	*Condylactis gigantea*	3.3%	4
6	*Trochus maculatus*	2.7%	1	*Calcinus elegans*	2.8%	4	*Clibanarius tricolor*	2.7%	2
7	*Nassarius venustus*	2.6%	1	*Trochus maculatus*	2.8%	2	*Stenopus hispidus*	2.6%	10
8	*Tectus fenestratus*	2.5%	2	*Stenopus hispidus*	2.7%	12	*Lysmata debelius*	2.5%	3
9	*Dardanus megistos*	2.4%	5	*Tectus fenestratus*	2.5%	3	*Entacmaea quadricolor*	2.4%	12
10	*Percnon gibbesi*	2.3%	5	*Tectus pyramis*	2.4%	4	*Dardanus megistos*	2.4%	6
11	*Tectus pyramis*	2.1%	2	*Percnon gibbesi*	2.3%	3	*Protoreaster nodosus*	2.3%	5
12	*Lysmata ankeri*	2.0%	1	*Condylactis gigantea*	2.1%	5	*Nassarius dorsatus*	2.2%	1
13	*Lysmata debelius*	2.0%	5	*Sabellastarte spectabilis*	1.7%	5	*Percnon gibbesi*	1.8%	5
14	*Condylactis gigantea*	1.9%	5	*Heteractis malu*	1.5%	6	*Nassarius venustus*	1.6%	1
15	*Sabellastarte spectabilis*	1.8%	4	*Lysmata ankeri*	1.5%	1	*Sabellastarte spectabilis*	1.6%	4
16	*Heteractis malu*	1.4%	6	*Protoreaster nodosus*	1.3%	4	*Nassarius distortus*	1.6%	1
17	*Calcinus elegans*	1.3%	3	*Engina mendicaria*	1.2%	2	*Heteractis malu*	1.5%	4
18	*Archaster typicus*	1.3%	6	*Entacmaea quadricolor*	1.1%	12	*Lysmata ankeri*	1.5%	1
19	*Engina mendicaria*	1.2%	2	*Nassarius distortus*	1.1%	1	*Pusiostoma mendicaria*	1.4%	2
20	*Stenorhynchus seticornis*	1.1%	5	*Archaster typicus*	1.1%	4	*Archaster typicus*	1.4%	6

### Species

More than half (52%) of the total fishes imported into the US (identified to species-level, Table 3) were represented by 20 species. There was a great deal of consistency within these top 20 species among the years of this study. The species ranking was identical between 2008 and 2009, and only the 20th ranked fish was different in 2011 (the blueband goby, *Valenciennea strigata*, replaced the royal gramma, *Gramma loreto*). The order of the top seven fish species was consistent across the years, and represented nearly 33% of total fish imports. The green chromis, *Chromis viridis*, was the most popular fish species across all three years (>10% of total fish imports) and was exported by 13–16 different countries, depending on the year. This *Chromis* species was unique in being collected from a large number of countries. The only other fish that was equally sourced from a large number of countries (an average of 15 per year) was the blue tang, *Paracanthurus hepatus*, (Table 3).

Invertebrates demonstrated a similar but more extreme trend. The top 20 species of invertebrates imported into the US were responsible for approximately 75% of total imports (identified to species-level, Table 3), yet there was more variability in the invertebrate top 20 species list compared to the fish list. Only the top two species (the scarlet hermit crab, *Paguristes cadenati*, and the scarlet skunk cleaner shrimp, *Lysmata amboinensis*) were consistently ranked across the three years. Overall, 25 invertebrate species were represented on the three yearly top 20 lists (Table 3).

**Figure 6:**
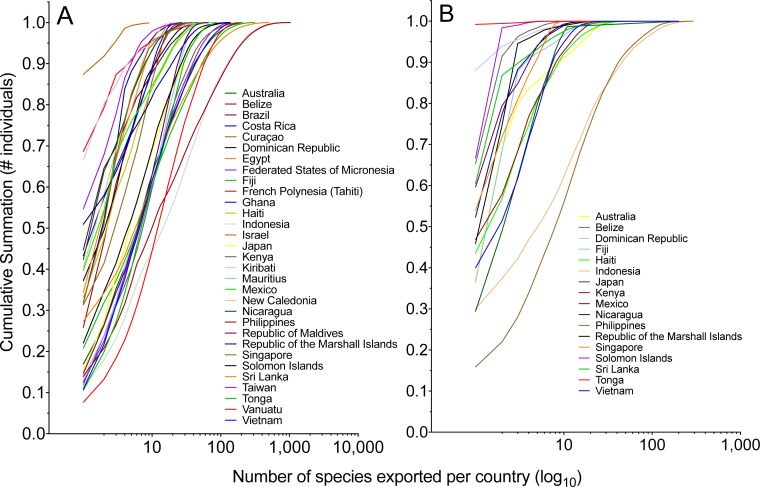
The cumulative summation of the number of (A) fishes and (B) invertebrates exported per country by rank order of species. The most-exported species represents a significant proportion of the total individuals exported per country, and this importance decreases as a country exports a greater number of species.

Each country could be represented by a single most exported species. Overall, the single most imported species averaged 37% (fishes) or 63% (invertebrates) of total species volume exported from that country (Tables 4 and 5). In general, countries that exported greater quantities of marine animals relied less on the contribution of the single most important species to export volume (Fig. 6).

**Table 4:** Top live aquarium fish species exported to the US from each exporting country (1°) with proportion of country’s export volume (individuals) (% Total) by year.

Export Country	2008		2009		2011	
	1°Species	% Total	1°Species	% Total	1°Species	% Total
Australia	*Amphiprion ocellaris*	42.3%	*Choerodon fasciatus*	14.7%	*Choerodon fasciatus*	16.9%
Belize	*Gramma loreto*	22.4%	*Gramma loreto*	27.0%	*Holacanthus ciliaris*	25.8%
Brazil	*Holacanthus ciliaris*	23.0%	*Holacanthus ciliaris*	28.6%	*Holacanthus ciliaris*	44.5%
Canada	*Eumicrotremus orbis*	76.9%	*Rhinoptera jayakari*	66.7%	*Eptatretus stoutii*	42.3%
Cook Islands	*Pseudanthias ventralis*	45.0%	*Pseudanthias ventralis*	46.0%	–	–
Costa Rica	*Thalassoma lucasanum*	31.1%	*Thalassoma lucasanum*	28.0%	*Elacatinus puncticulatus*	28.1%
Curaçao	*Elacatinus genie*	28.0%	*Liopropoma carmabi*	18.6%	*Gramma loreto*	31.2%
Dominican Republic	*Gramma loreto*	59.8%	*Gramma loreto*	60.4%	*Gramma loreto*	36.0%
Egypt	–	–	–	–	*Zebrasoma xanthurum*	31.7%
Eritrea	*Zebrasoma xanthurum*	23.2%	*Zebrasoma xanthurum*	24.6%	–	–
Fed. States of Micronesia		–	–	–	*Pseudanthias bartlettorum*	12.3%
Fiji	*Pseudanthias squamipinnis*	9.6%	*Pseudanthias squamipinnis*	12.2%	*Chromis viridis*	20.3%
French Polynesia (Tahiti)	*Neocirrhites armatus*	43.9%	*Neocirrhites armatus*	64.6%	*Neocirrhites armatus*	68.0%
Ghana	*Balistes punctatus*	20.2%	*Balistes punctatus*	36.3%	*Holacanthus africanus*	41.4%
Guatemala	*Selene brevoortii*	58.8%	*Selene brevoortii*	64.7%	–	–
Haiti	*Gramma loreto*	33.8%	*Gramma loreto*	31.2%	*Gramma loreto*	32.9%
Hong Kong	*Zebrasoma flavescens*	76.3%	*Dascyllus trimaculatus*	40.0%	*Chordata*	99.7%
Indonesia	*Chromis viridis*	10.5%	*Chromis viridis*	8.9%	*Chromis viridis*	10.8%
Israel	–	–	*Premnas biaculeatus*	34.7%	*Amphiprion ocellaris*	88.7%
Japan	*Parapriacanthus ransonneti*	66.2%	*Parapriacanthus ransonneti*	13.5%	*Paracentropogon rubripinnis*	14.2%
Kenya	*Labroides dimidiatus*	15.5%	*Labroides dimidiatus*	14.2%	*Labroides dimidiatus*	12.0%
Kiribati	*Centropyge loricula*	70.1%	*Centropyge loricula*	63.2%	*Centropyge loricula*	65.1%
Malaysia	*Amphiprion ocellaris*	35.7%	–	–	*Paracanthurus hepatus*	100.0%
Mauritius	*Amphiprion chrysogaster*	12.0%	–	–	*Macropharyngodon bipartitus*	13.2%
Mexico	*Holacanthus passer*	49.5%	*Holacanthus passer*	35.8%	*Holacanthus passer*	39.0%
Netherlands Antilles	*Elacatinus genie*	58.9%	–	–	–	–
New Caledonia	*Chaetodontoplus conspicillatus*	84.5%	*Chaetodontoplus conspicillatus*	85.3%	*Cirrhilabrus laboutei*	40.3%
Nicaragua	*Apogon retrosella*	22.4%	–	–	*Acanthemblemaria hancocki*	39.5%
Papua New Guinea	*Amphiprion percula*	29.7%	*Paracanthurus hepatus*	23.4%	–	–
Philippines	*Chromis viridis*	11.7%	*Chromis viridis*	13.3%	*Chromis viridis*	13.6%
Rep. of Maldives	*Acanthurus leucosternon*	12.9%	*Acanthurus leucosternon*	12.7%	*Acanthurus leucosternon*	14.7%
Rep. of the Marshall Islands	*Centropyge loricula*	53.7%	*Centropyge loricula*	41.9%	*Centropyge loricula*	40.1%
Saudi Arabia	*Dascyllus marginatus*	30.7%	*Anampses caeruleopunctatus*	36.8%	–	–
Singapore	*Amphiprion ocellaris*	42.3%	*Amphiprion ocellaris*	73.0%	*Amphiprion percula*	31.6%
Solomon Islands	*Paracanthurus hepatus*	19.4%	*Paracanthurus hepatus*	33.7%	*Paracanthurus hepatus*	20.4%
Sri Lanka	*Valenciennea puellaris*	22.5%	*Valenciennea puellaris*	21.1%	*Valenciennea puellaris*	26.8%
Taiwan	*Pomacanthus maculosus*	24.8%	*Pomacanthus maculosus*	19.4%	*Amphiprion ocellaris*	55.2%
Thailand	*Amphiprion ocellaris*	87.8%	*Amphiprion ocellaris*	90.5%	–	–
The Bahamas	*Haemulon sciurus*	17.5%	*Chromis cyanea*	11.5%	*Haemulon flavolineatum*	88.9%
Tonga	*Centropyge bispinosa*	14.8%	*Centropyge bispinosa*	12.7%	*Meiacanthus atrodorsalis*	9.6%
United Arab Emirates	–	–	–	–	*Zebrasoma xanthurum*	63.6%
United Kingdom	*Amphiprion ocellaris*	76.5%	–	–	–	–
Vanuatu	*Centropyge loricula*	9.4%	*Chrysiptera rollandi*	12.8%	*Chromis viridis*	6.9%
Vietnam	*Nemateleotris magnifica*	8.3%	*Nemateleotris magnifica*	16.7%	*Chaetodontoplus septentrionalis*	12.3%
Yemen	*Zebrasoma xanthurum*	48.2%	*Zebrasoma xanthurum*	47.6%	–	–
Total	*Chromis viridis*	10.0%	*Chromis viridis*	10.2%	*Chromis viridis*	11.2%

**Table 5:** Top live aquarium invertebrate species exported to the US from each exporting country (1°) with proportion of country’s export volume (individuals) (% Total) by year.

Export country	**2008**		**2009**		**2011**	
	1°Species	% Total	1°Species	% Total	1°Species	% Total
Australia	*Pseudocolochirus violaceus*	75.6%	*Actinia tenebrosa*	46.1%	*Actinia tenebrosa*	37.2%
Belize	*Mithraculus sculptus*	72.5%	*Mithraculus sculptus*	51.9%	*Mithraculus sculptus*	65.3%
Canada	*Enteroctopus dofleini*	100.0%	–	–	–	–
China	*Actinia equina*	70.5%	–	–	–	–
Costa Rica	–	–	*Lysmata wurdemanni*	78.1%	–	–
Curaçao	–	–	*Lysmata wurdemanni*	100.0%	*Paguristes cadenati*	94.7%
Dominican Republic	*Paguristes cadenati*	92.2%	*Paguristes cadenati*	95.1%	*Paguristes cadenati*	88.0%
Fiji	*Linckia laevigata*	78.0%	*Linckia laevigata*	87.0%	*Linckia laevigata*	36.5%
French Polynesia (Tahiti)	–	–	*Holothuria (Halodeima) atra*	52.2%	–	–
Ghana	*Actinia tenebrosa*	85.3%	*Actinia equina*	94.8%	*Lysmata grabhami*	99.5%
Haiti	*Paguristes cadenati*	46.3%	*Paguristes cadenati*	47.1%	*Paguristes cadenati*	43.6%
Hong Kong	*Entacmaea quadricolor*	100.0%	–	–	*Entacmaea quadricolor*	100.0%
Indonesia	*Trochus maculatus*	19.3%	*Trochus maculatus*	19.1%	*Trochus maculatus*	30.3%
Japan	*Aurelia aurita*	52.9%	*Aurelia aurita*	58.7%	*Catostylus mosaicus*	29.4%
Kenya	*Lysmata amboinensis*	56.0%	*Lysmata amboinensis*	69.0%	*Lysmata amboinensis*	48.9%
Kiribati	–	–	–	–	*Panulirus versicolor*	100.0%
Mauritius	*Heteractis magnifica*	100.0%	–	–	–	–
Mexico	*Pentaceraster cumingi*	74.9%	*Turbo fluctuosus*	40.1%	*Dardanus megistos*	59.7%
Nicaragua	*Turbo fluctuosus*	49.2%	–	–	*Coenobita clypeatus*	52.3%
Papua New Guinea	*Archaster typicus*	45.7%	*Archaster typicus*	36.5%	–	–
Philippines	*Lysmata amboinensis*	15.9%	*Lysmata amboinensis*	18.8%	*Lysmata amboinensis*	16.3%
Rep. of Maldives	*Echinaster (Echinaster) sepositus*	50.0%	*Echinaster (Echinaster) sepositus*	83.3%	*Pusiostoma mendicaria*	45.9%
Rep. of the Marshall Islands	*Engina mendicaria*	70.8%	*Calcinus elegans*	45.2%	–	–
Singapore	*Tectus niloticus*	41.1%	*Entacmaea quadricolor*	34.7%	*Sabellastarte spectabilis*	54.0%
Solomon Islands	*Archaster typicus*	46.4%	*Archaster typicus*	65.8%	*Archaster typicus*	66.7%
Sri Lanka	*Lysmata amboinensis*	61.9%	*Lysmata amboinensis*	63.7%	*Lysmata amboinensis*	60.7%
Thailand	*Gecarcoidea natalis*	100.0%	–	–	–	–
The Bahamas	*Ophiocoma alexandri*	46.7%	*Ancylomenes pedersoni*	22.7%	–	–
Tonga	*Nassarius venustus*	92.0%	*Nassarius venustus*	77.6%	*Nassarius venustus*	99.2%
United Kingdom	–	–	–	–	*Chrysaora quinquecirrha*	50.0%
Vanuatu	*Linckia multifora*	28.2%	*Entacmaea quadricolor*	56.4%	*Entacmaea quadricolor*	54.3%
Vietnam	*Macrodactyla doreensis*	34.1%	*Macrodactyla doreensis*	36.5%	*Entacmaea quadricolor*	40.1%
Total	*Paguristes cadenati*	22.1%	*Paguristes cadenati*	20.9%	*Paguristes cadenati*	14.3%

### Comparison to LEMIS data

**Figure 7:**
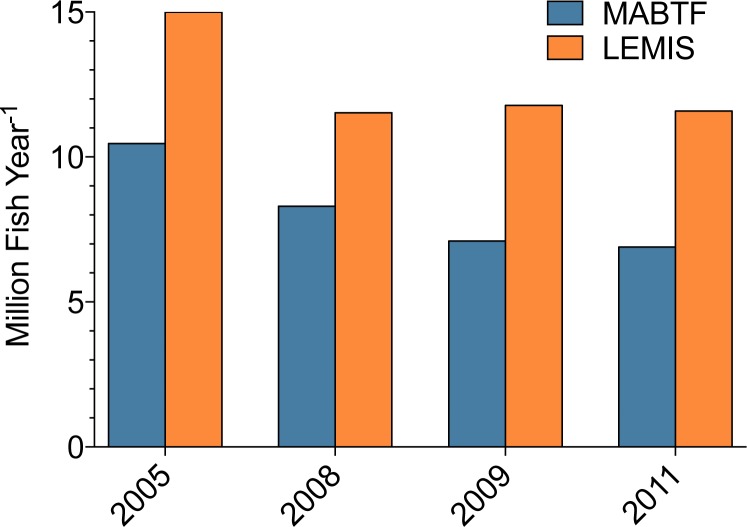
Comparison of total number of marine aquarium fish imports into the US. Comparison of total number of marine aquarium fish imports into the US according to the Law Enforcement Management Information System (LEMIS) and The Marine Aquarium Biodiversity and Trade Flow (MABTF) online database across 4 years. Data from 2008, 2009, and 2011 is from the dataset presented here, and data from 2005 was presented in Rhyne et al. (2012).

The LEMIS database contains information regarding non-CITES-listed species recorded on declarations under general codes. LEMIS data is produced by US-based importers from shipment declarations, where importers input shipment data into the required 3-177 declaration form and present the completed shipment declaration with corresponding invoice to USFWS prior to shipment clearance. We have demonstrated elsewhere (Rhyne et al., 2012) that this method of gathering import data is fraught with errors; first, importers commonly mislabel shipments as containing marine aquarium species when they only contain freshwater fish, non-marine species, or non-aquarium fish (all increasing the total number of fish reported in the LEMIS database); second, the data do not appear to be updated if shipments are canceled or modified (there is sometimes a significant mismatch between the number of individuals on the declaration and the corresponding values on the invoices); third, importers commonly misrepresent the country of origin and source (wild-caught/captive-bred) of species in shipments. As previously discussed (Rhyne & Tlusty, 2012), LEMIS is a tool designed for internal use by USFWS, primarily relating to volume of boxes arriving at ports and CITES compliance. Shipments of non-CITES-listed species and/or unregulated species are not held to any data integrity standards. We propose that the invoice-based method of data collection presented here can rectify many of the data deficiency issues that currently exist within the marine aquarium trade. Through this work, it was observed that the numbers of fishes imported into the US were routinely 60%–72% fewer than the import volumes reported by the LEMIS database (Fig. 7). A large proportion of the declaration form overestimate was a result of importers misclassifying shipments as MATF when they only contained freshwater species. Occasionally, entire freshwater shipments were erroneously listed as MATF. A second unknown portion of this error was missing invoices. Not all invoices were recovered from the LEMIS system. Several hundred records were either missing the invoice or exhibited invoice/declaration mismatch, making the data impossible to verify. Similarly, invoice-based data reported a total of 45 countries exporting MATF, which was only 60% of the 76 export countries reported by the LEMIS database (Table 6). These countries represented 5%, 6%, and 11% of the total volume of MATF imported into the US according to the LEMIS database during 2008, 2009, and 2011, respectively (Table 6). Third is that the declaration is typically completed days before the order is packed, which invites variation between estimated and actual order volume. Finally, there was a lack of adherence to differentiating “wild-caught” and “captive-bred” animals (Rhyne, Tlusty & Kaufman, 2012). The case studies presented below use the invoice-based dataset to shed light on this discrepancy.

### Estimated fish

In October of 2000, 810,705 fishes and 124,308 invertebrates were imported. During the years 2008, 2009 and 2011, October represented on average 8.7% of fish and 8.6% of invertebrate imports into the US. Thus, it can be estimated that 9,327,754 fish and 1,442,859 invertebrates were imported into the US during calendar year 2000. Following this example, 10,766,706 and 11,229,443 fish were imported into the US in 2004 and 2005 respectively, no invertebrate data was available for 2004 and 2005 data.

**Table 6:** Countries reported in LEMIS database that export live aquarium fishes to the US. Data include number of individuals exported (LEMIS (No.)) and proportion of LEMIS invoices recovered in the present study (Invoice (%)). Shaded countries are those represented in the invoice-based assessment of fish imports to the US.

Country	2008		2009		2011		2008–2011	
	LEMIS (No.)	Invoice (%)	LEMIS (No.)	Invoice (%)	LEMIS (No.)	Invoice (%)	LEMIS (No.)	Invoice (%)
Antarctica	–	–	–	–	49	–	49	–
Australia	16,908	76.2	13,973	62.8	10,545	90.8	41,426	75.4
Barbados	–	–	–	–	82	–	82	
Belgium	266	–	–	–	–	–	266	–
Belize	19,957	47.5	18,214	48.6	27,840	53.8	66,011	50.5
Brazil	61,247	14.3	15,342	21.8	3,179	63.1	79,768	17.7
Canada	53	98.1	119	2.5	56	46.4	228	35.5
Cayman Islands	14	–	–	–	–	–	14	–
China	354,093	–	126,218	–	44,151	–	524,462	–
Christmas Island	–	–	–	–	1,712	–	1,712	–
Colombia	24,386	–	–	–	12,594	–	36,980	–
Dem. Rep. of the Gongo	–	–	185	–	–	–	185	–
Cook Islands	4,793	99.4	3,330	99.6	–	–	8,123	99.5
Costa Rica	38,904	20.3	15,770	22.4	6,220	98.7	60,894	28.9
Cuba	20	–	–	–	–	–	20	–
Curacao	–	–	–	–	2,992	112.5	2,992	176.4
Czech Republic	1,154	–	428,022	–	80,500	–	509,676	–
Dominican Republic	58,497	37.8	64,254	45	39,687	86.4	162,438	57.8
Ecuador	–	–	5,990	–	–	–	5,990	–
Egypt	–	–	–	–	755	126.2	755	126.2
Eritrea	2,796	340.0	3,046	130.9	–	–	5,842	231.4
Fed. States of Micronesia	–	–	–	–	5,515	100.6	5,515	100.6
Fiji	13,8801	83.2	119,535	73.9	171,364	91.4	429,700	84.3
French Polynesia (Tahiti)	50,261	85.2	30,789	98.0	30,914	93.8	111,964	91.3
Ghana	1,119	45.5	982	69.9	808	87.6	2,909	66.4
Guatemala	7,755	13.6	343	100.0	–	–	8,098	17.3
Guinea	357	–	–	–	–	–	357	–
Haiti	306,084	78.6	280,196	77.1	135,890	93.3	722,170	81.5
Hong Kong	205,408	0.1	25,806	0	141,888	11.6	373,102	4.5
India	5,374	–	4,932	–	–	–	10,306	–
Indonesia	3,044,574	78.9	2,743,170	72.8	2,228,446	83.8	8,016,190	79.0
Israel	22	–	818	81.4	25,990	84.6	26,830	84.4
Japan	1,911	59.3	1,790	63.5	820	69.4	4,521	62.8
Kenya	175,607	82.1	178,715	77.8	123,248	82.7	477,570	81.3
Kiribati	143,615	85.6	93,586	84.2	118,164	89.4	355,365	86.9
Republic of Korea	–	–	–	–	6	–	6	–
Malaysia	6,516	9.5	11,937	–	15,255	0.1	33,708	1.9
Mauritania	184	–	–	–	–	–	184	–
Mauritius	6,465	12.7	–	–	681	99.9	7,146	21.0
Mexico	5,147	100.5	15,805	80.3	22,331	67.8	43,283	77.4
Morocco	141	–	–	–	–	–	141	–
Netherlands	–	–	87	–	175	–	262	–
Netherlands Antilles	2,178	14.6	1,558	–	628	–	4,364	7.3
New Caledonia	317	26.5	86	87.2	617	62.7	1,020	53.5
New Zealand	–	–	–	–	1	–	1	–
Nicaragua	15,647	57.4	–	–	2,120	87.1	17,767	61.0
Nigeria	4,005	–	4,249	–	9,446	–	17,700	–
Norway	196	–	2,853	–	2,903	–	5,952	–
Papua New Guinea	11,899	57.3	13,167	63.1	–	–	25,066	60.8
Peru	80,963	–	67,609	–	10,395	–	158,967	–
Philippines	5,504,928	85.3	4,815,066	83.6	4,545,933	85.8	14,865,927	85.6
Rep. of Maldives	27,215	90.3	23,572	93.7	37,423	91.8	88,210	92.1
Rep. of the Marshall Isl	50,143	75.7	163,433	70.8	152,000	93.5	365,576	91.4
Saudi Arabia	7,304	4.5	2,131	0.9	–	–	9,435	3.7
Sierra Leone	244	–	–	–	–	–	244	–
Singapore	310,090	0.8	279,794	0.9	325,715	4.3	915,599	2.1
Slovakia	119	–	–	–	–	–	119	–
Solomon Islands	66,057	71.5	58,397	59.5	42,562	97.9	167,016	75.2
Sri Lanka	337,521	60.0	1,807,583	12	1,981,399	10.7	4,126,503	15.5
Suriname	–	–	214	–	–	–	214	–
Taiwan	54,623	2.8	47,430	1.9	6,950	35.2	109,003	4.6
United Republic of Tanzania	253	–	–	–	–	–	253	–
Thailand	207,582	19.2	115,952	7.2	1,117,626	–	1,441,160	3.3
The Bahamas	1,557	61.1	1,524	28.3	1,397	21.3	4,478	37.5
Tonga	57,120	48.8	13,787	58.4	2,474	108.2	73,381	53.5
Trinidad and Tobago	–	–	107,805	–	46,360	–	154,165	–
Tunisia	558	–	–	–	–	–	558	–
Ukraine	93	–	–	–	–	–	93	–
United Arab Emirates	–	–	–	–	79	97.5	79	97.5
United Kingdom	3,721	99.7	583	–	4	–	4,308	86.1
United States	828	–	87	–	45	–	960	–
Vanuatu	22,850	86.2	15,538	81.5	15,924	90.5	54,312	86.9
Various States	31,771	–	13,369	–	23,350	–	68,490	–
Vietnam	26,621	54.8	10,832	60.4	9,597	50.3	47,050	55.3
Yemen	20,230	51.1	13,114	90.5	–	–	33,344	66.6
Zambia	–	–	1,286	–	–	–	1,286	–
**Total (No Invoice Data)**	**50,5041**	**–**	**776,984**	**–**	**1,350,027**	**–**	**1,499,694**	**–**
**Total (All Countries)**	**11,529,062**	**72.0**	**11,783,973**	**60.3**	**11,586,805**	**59.5**	**34,899,840**	**64.6**

### Confusion Between “Wild” and “Cultured” Production

**Figure 8:**
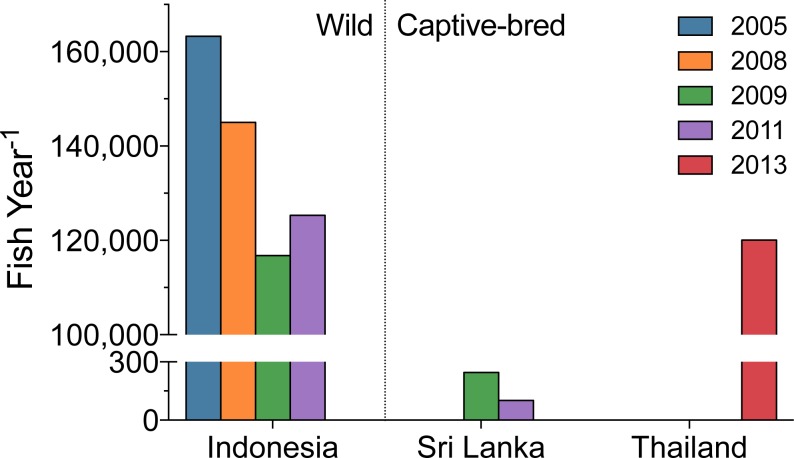
Annual volume of Bangaii cardinalfish (*Pterapogon kauderni*) into the US by top export countries. Exports from Sri Lanka (2009, 2011) and Thailand (2013) illustrate the likely prevalence of previously unrecognized captive-bred *P. kauderni* in the trade.

The Banggai cardinalfish, *Pterapogon kauderni*, is a popular marine fish in the aquarium trade (ranked the 10th, 11th, and 8th most imported fish into the US during 2008, 2009, and 2011, Table 5). It was one of the original marine aquarium aquaculture success stories (Talbot et al., 2013; Tlusty, 2002), which was supposed to reduce the need for wild-caught fishes. While aquaculture should dominate the source of the fish, all *P. kauderni* imported during this three-year span were reported as wild-caught fish. Yet import records from Sri Lanka in 2009 and 2011, and Thailand in 2013 (both outside the natural geographic range of *P. kauderni*) suggest this is not the case (Fig. 8).

**Figure 9:**
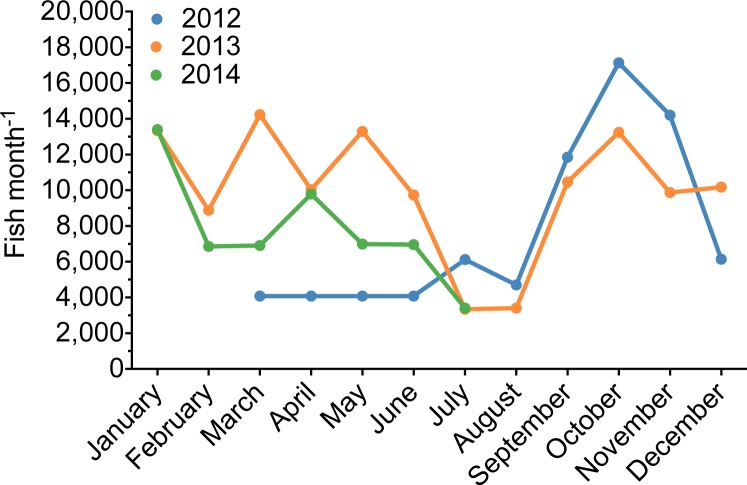
Temporal variability of the volume of captive-bred Bangaii cardinalfish (*Pterapogon kauderni*) imported into the US. Temporal variability of the volume of assumed captive-bred Bangaii cardinalfish (*Pterapogon kauderni*) imported into Los Angeles, California, US. This seasonal variability is consistent with the import of wild fish.

The export volume of *P. kauderni* from Thailand followed the typical aquarium trade pattern of lower volumes exported in the summer months (June–August) and in December (Fig. 9). Interestingly in 2013, the only year with a 12-month data set starting in January and ending in December, the volume of *P. kauderni* (∼120,000 individuals/year) was approximately 75% of the average total import volume of this species recorded per year for 2008, 2009 or 2011. Further, these fish were listed on import declarations as ranging in size from 1 to 1.5 inches. A 1-inch fish is smaller than the average wild-caught fish (A Rhyne, pers. obs., 2013) and instead represents the typical shipping size of a captive-bred fish. Shipment manifests also list the number of Dead On Arrival (DOA) from previous shipments, which were extremely low (<0.5%) for wild-caught *P. kauderni*.

The shipment manifests have common errors that can be observed on the 3–177 USFWS declarations. On several occasions the importer incorrectly indicated that shipments were wild-caught animals (“W”). After examining the invoices and associated documents, (i.e., health and aquaculture certificates), we determined that all shipments of *P. kauderni* from Thailand to Quality Marine during the period examined were captive-bred (“C”), and the importer erroneously selected “W” in the Source box (Box 18B, 3–177 form). Given the current Endangered Species Act (ESA) listing for *P. kauderni* (NOAA, 2014), accurate and timely trade data are crucial to the sustainable management of this species.

### Misidentification and Unknown Trade

Similar to the Banggai cardinalfish, clownfishes exported from Southeast Asia are commonly labeled as wild-caught while many are in fact captive-bred. This inaccuracy is compounded by the misidentification of clownfishes on export invoices, especially among species with similar morphological appearances (e.g., the orange clownfish, *Amphiprion percula*, and the common clownfish, *A. ocellaris*). Use of http://www.aquariumtradedata.org not only sheds light on source-errors (as seen in the Banggai cardinalfish case study) but also on potential species misidentifications.

**Figure 10:**
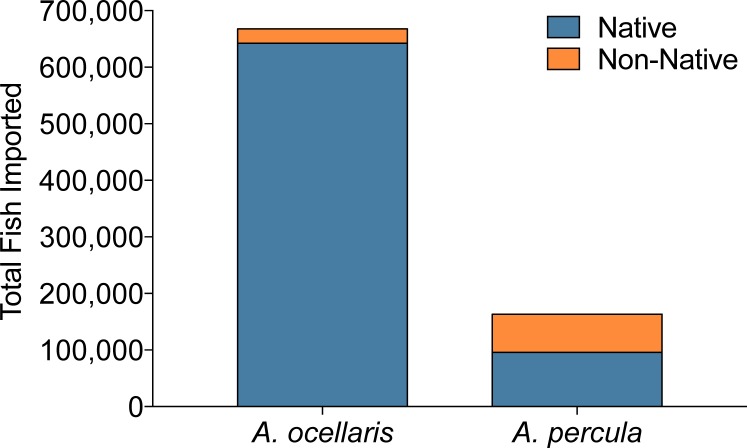
Imports of orange clownfish (*Amphiprion ocellaris*/*A. percula*) into the US aggregated over 2008, 2009, and 2011. Export countries were grouped based on the documented geographic range of each species. All non-native individuals are either actually native (but of an unknown distribution), captive-bred, or misidentified as to origin on the shipping invoice.

Recently, the orange clownfish (*A. percula*) was proposed to be listed as threatened or endangered under the ESA, mainly due to its small geographic distribution and obligate relationship with giant sea anemones prone to bleaching events in the Coral Triangle. However, the proposition was also based on the assumption that out of the 400,000 individuals from the *percula*/*ocellaris* complex imported into the US in 2005 (Rhyne et al., 2012), (a) all specimens were wild-caught, and (b) *A. percula* and *A. ocellaris* were equally traded, with 200,000 individuals of each species being harvested. Utilization of http://www.aquariumtradedata.org can help clarify issues regarding origination of these species. While in 2008, 2009 and 2011, 831,398 individual clownfishes of the *percula*/*ocellaris* complex were imported into the US (Fig. 10), only 163,547 individuals were *A. percula* (24.5%, Fig. 10). These data suggest that the original assumptions of trade volume used to petition for ESA listing were strongly overestimated.

Further, the Countries of Origin feature of the database revealed that of the ten export countries of *A. percula*, seven countries (41% of all individuals) fall outside the natural geographic range of this species (Fautin & Allen, 1997; Froese & Pauly, 2015). Furthermore, five of the seven non-native locations are established producers of captive-bred *A. percula*. Based on this, 7% of the non-native individuals are likely captive-bred specimens. The remaining two non-native countries (Singapore and the Philippines) account for the residual 93% of the non-native individuals and are likely misidentifications. Interestingly, both Singapore and the Philippines fall within the natural geographic range of *A. ocellaris*, which is commonly confused with *A. percula*. While these individuals may be misidentified, it is also important to note that Singapore is a known trans-shipping country, and could have imported their specimens from another country, making the true origin of these specimens unattainable. Furthermore, Singapore is a leader in captive-bred production of aquarium fish, and thus many clownfish could be of captive-bred origin.

In summary, 41% of *A. percula* imported into the US over the three-year span (a) were misidentified as to species or export country, (b) were misidentified as to source (wild-caught versus captive-bred), or (c) represent a recently expanded home range not yet noted within the scientific literature (unlikely). Regardless of the reason, the contribution of *A. percula* imports to the *percula*/*ocellaris* complex is not only substantially less than assumed, but also is likely even lower based on the high percent of geographic anomalies reported here. Ultimately, this case study confirms the need for more accurate and detailed trade data, such as that provided via http://www.aquariumtradedata.org, for any potential ESA listing activity.

The orange clownfish case study demonstrates the effect that species-level nomenclature confusion can have on trade data. Users of http://www.aquariumtradedata.org have illuminated other examples of species misidentification, such as *Centropyge argi*, an Atlantic species (Froese & Pauly, 2015) with a significant volume of reported exports from Indonesia. While species-level confusion may be common for specious genera of fishes such as *Centropyge* and *Amphiprion*, it is important to recognize instances of more blatant misidentification. For example, the iconic yellow tang, *Zebrasoma flavescens*, although a Hawaiian endemic, appears in this database as being exported from 13 countries; it is difficult to imagine this fish being confused with anything else. Continuous biogeographical filtering of invoice data by website users will help to minimize misidentification errors. One of the egregious cases of identification error is invoice items listed as “unknown”. These must find a home in the database, and at this point in time, are ultimately listed as “Chordata.” This lack of identification of fishes raises the issue that different countries have various reporting requirements that can create data deficiencies within the database. If one queries exports from Hong Kong, there are no reporting requirements, and thus all fish are entered into the database as the generic category “Chordata.” A deficiency of reporting requirements for this database is the lack of between-country agreement.

## Discussion

The deficiency of comprehensive and overarching data relating to the global marine aquarium trade hinders progress toward its effective management (Foster, Wiswedel & Vincent, 2016; Fujita et al., 2014; Rhyne et al., 2012). We believe that access to more accurate data will allow for increased public engagement in trade sustainability and guide responsible trade management. These data can stimulate action toward consumer education, address challenges such as misidentification, and better manage species. Ideally these will result in greater sustainability (Tlusty et al., 2013). Currently, there is no overarching system for tracking species-level import/export data for the marine aquarium trade. This is exacerbated by the lack of standard recordkeeping between different countries (Green, 2003). Coupled with this is the fact that present data systems are either overly general, based on declaration forms (LEMIS), or specific to the trade of rare and threatened species (CITES, Foster, Wiswedel & Vincent, 2016). The Global Marine Aquarium Database (Green, 2003) attempted to make sense of some of these discrepancies, but can be difficult to use due to its data structures and relational databases. These complications and data limitations promote misinterpretation, as evidenced by the trade volume under- and over-estimates discussed here. Changes to and standardization of the way live animal trade data are recorded are necessary to accurately assess trade pathways and data inaccuracies. This will avoid misinterpretations with potentially costly consequences of social, economic, and ecological proportions (e.g., the use of such data to affect ESA listing status). Such costs will only be exacerbated as the aquarium industry continues to grow.

Capturing invoice-based data can waylay many of the deficiencies of the extant databases and should prove useful to both conservation organizations and government agencies by helping to address the aquarium trade data deficiency that currently exists. The benefit of the more detailed invoice data we focused on here is that it allowed for a truer estimate of aquatic wildlife trade. The recent ESA petitions for both the Banggai cardinalfish (NOAA, 2014) and the orange clownfish (Maison & Graham, 2015) were proposed based on incorrect trade data and, in each of these cases, we demonstrated that increased knowledge of production areas and modalities do not support the underlining trade assumptions of these petitions. The assumptions of these ESA listings were demonstrated to be erroneous in part due to reported source (wild-caught, captive-bred, farmed) inaccuracies of shipped animals. For example, exporters will often mark farmed corals as wild corals, even when they have proper CITES permits for the export of farmed corals (Rhyne, Tlusty & Kaufman, 2014). Many exporters do not have the appropriate paperwork or government support needed to accurately mark corals as captive-bred or farmed on CITES documents, often because of the onerous process required to certify that corals are of farmed origin. While improved analysis of invoices will help limit some of this misreporting, it will not be totally unabated until a full fishery/farm to retail traceability program is initiated.

Further issues with the clownfish ESA listing occurred because of demonstrated geographic misidentification. Seven of the ten export countries of the orange clownfish fell outside the natural geographic range of this species. Even though these can be indicated as erroneous data, correcting these anomalies is outside the purview of http://www.aquariumtradedata.org, as it was deemed important to record data as indicated on the invoice. Further discussion of discrepancy can occur through in-depth analysis of these data and such efforts are welcomed. As this database develops, it will be important to identify the commonly misidentified species, and to help the entire trade improve its species documentation. Ideally, lists of ‘look-alike’ species can be created, and machine learning can be used to derive biogeographically edited species lists. One of the critical assumptions of this work is that the invoice represents the true contents of the shipment. There are a number of reasons this may not be the case, including but not limited to miscounting, misidentification, and intentional substitution. The question of invoice veracity has been highlighted by others (Jones, 2008; Wabnitz et al., 2003), and without a study directly comparing invoice to box contents, the exact correlation will remain unknown. If it is assumed that the trade is based on above-board honest business practices, then the invoice should be an accurate representation of the trade. However, the greater the dishonesty in the trade (e.g., invoicing a shipment of corals as MATF), the greater the disconnect between invoice and box contents. This project was recently named a Grand Prize Winner of the Wildlife Crime Tech Challenge (www.wildlifecrimetech.org), which will spur continued development of this project, with an emphasis on increasing honest business practices by more easily identifying and enforcing illegal and inaccurate trade activity.

The development of http://www.aquariumtradedata.org is a first step toward improving the data, which will allow for better management and oversight of the trade in marine aquatic animals. However, the invoice analysis was developed from a post-import standpoint. The shipments were accepted at import, the paperwork processed, and the invoices stored, only to be recovered from storage and delivered for analysis within this program. However, the OCR data processing has the potential to be utilized in real time. This would allow for shipment diagnostics to be conducted, which could potentially identify misidentified or even illegal shipments. Such an import risk-based screen tool exists under the FDA’s Predictive Risk-based Evaluation for Dynamic Import Compliance Targeting program (http://www.fda.gov/ForIndustry/ImportProgram/ucm172743.htm), and we propose that a similar model would be effective for the aquatic wildlife trade. Ultimately, such an analysis would provide support to port agents to help them more effectively monitor the trade.

Aquaculture is a significant means of fish production (Tlusty, 2002). This will present a challenge because the lack of reporting for domestic sales will obscure patterns in the trade. Murray & Watson (2014) observed clownfish to be the most popular species kept by aquarists, although they were not the most popular species imported in our database. The US domestic production will obscure the relationship between fishes imported, and those actually being kept by hobbyists. However, we need to step towards greater recordkeeping on species in the trade, and while we focus on international trade, it would be ideal if records of domestic production could additionally be kept.

While it was not implicitly necessary to estimate the number of individuals imported for years of incomplete data (2000, 2004 and 2005), incomplete data in 2004 and 2005 compared to complete data in the later years could give the impression the trade was increasing. A common query without the estimated number of fish would result in a figure where the total number of indivudals in 2000 was 8% while 2004 and 2005 data would be approximately half that of 2008, 2009 and 2011. Therefore, the estimated fish numbers were calculated to create a more cohesive visual presentation of data, and to avoid the incorrect analysis that numbers of US imports of marine aquarium fishes and invertebrates are increasing. Prior analysis indicated the trade has decreased from its peak in 2005 following the economic recession and a shift to smaller tank sizes (Rhyne & Tlusty, 2012; Rhyne, Tlusty & Kaufman, 2012). Estimated data should be treated with caution given their (by definition) degree of error. We encourage users of this database to determine if more sufficient methods to estimate unknown data can be validated.

In summary, wildlife data tracking systems require improvement (Chan et al., 2015; Foster, Wiswedel & Vincent, 2016); we are beyond the age of tracking animal shipment volume solely for the purpose of assessing port agent staffing needs. The systems currently in place for tracking non-CITES-listed aquarium animals have proven ineffective in producing meaningful data that can move the trade toward sustainability and conservation (Vincent et al., 2014). The invoice-based dataset presented here, while set up as a post-import assessment tool, could be easily modified into a real-time aquarium trade data monitoring system. Ideally this can have a positive impact on increasing the veracity of the LEMIS system. It behooves the largest aquatic wildlife importer in the world to showcase real constructive intent to foster sustainability in the trade and to create positive change in an important industry. This proof that the trade can be passivily monitored (Wallace et al., 2014) is a huge step toward driving positive change in the trade. The next step will be to monitor aquarium trade pathways in real-time, as this is crucial to effectively assist the management of the trade of marine aquarium wildlife for the home aquarium industry. A goal for real-time, simultaenous monitoring of exports and imports is now within reach.

## Additional Information and Declarations

### Data Availability

The following information was supplied regarding data availability: Marine Aquarium Biodiversity and Trade Flow online database: https://www.aquariumtradedata.org. This site acts as the public repository for the data, it is graphically displayed with CSV download option.
